# Mapping of uterine-related neurons in central nervous system of mice by *trans*-synaptic tracing with pseudorabies virus

**DOI:** 10.1016/j.bbrep.2026.102630

**Published:** 2026-05-17

**Authors:** Hehua Wang, Jinqiong Zhan, Qingyue Cao, Jingjing Hu, Fen Liu, Yan Wang, Tong Dai, Xia Yang, Qinyu Yang, Pengcheng Huang, Chunhua Tu

**Affiliations:** a Department of Obstetrics and Gynecology, The First Affiliated Hospital, Jiangxi Medical College, Nanchang University, Nanchang, Jiangxi, 330006, PR China; b Jiangxi Mental Hospital & Affiliated Mental Hospital, Jiangxi Medical College, Nanchang University, Nanchang, Jiangxi, 330029, PR China; c Nanchang City Key Laboratory of Biological Psychiatry, Health Commission of Jiangxi Province Key Laboratory of Psychiatry, Jiangxi Provincial Clinical Research Center on Mental Disorders, Jiangxi Mental Hospital, Nanchang, Jiangxi, 330029, PR China; d Department of Neurology, The First Affiliated Hospital, Jiangxi Medical College, Nanchang University, Nanchang, Jiangxi, 330006, PR China

**Keywords:** Uterus, Pseudorabies virus tracing, Neural circuit, Oxytocin, Olfactory

## Abstract

The uterus, a key organ for maintaining reproductive health and fertility, is precisely regulated by central nervous system (CNS). This study employed multimodal neural tracing and c-Fos staining to investigate the multisynaptic pathways connecting uterus and CNS in mice, as well as the potential neural mechanisms through which olfactory stimuli influence uterine activity. First, the PRV-CAG-EGFP was injected into uterine myometrium. PRV-infected neurons were observed to ascend over time from spinal cord and medulla oblongata to forebrain, ultimately appearing in regulatory nuclei such as paraventricular nucleus of hypothalamus (PVN), medial amygdala (MeA) and central amygdala (CeA), confirming the multilevel neural connections from uterus to CNS. Further anatomical tracing identified connection between olfactory/accessory olfactory bulbs and MeA, but not CeA, suggesting that MeA may act as a crucial relay for olfactory information transmission. In odorant screening, butyric acid (BA) activated olfactory bulb (OB), PVN, MeA, and CeA. This effect was abolished following nasal mucosal injury, indicating its dependence on an intact olfactory pathway. Additionally, BA also activated a subset of oxytocin-positive neurons within PVN. Co-labeling experiments combining PRV tracing and c-Fos staining identified neurons in PVN, CeA, and MeA that were both activated by BA and *trans*-synaptically connected to uterus. Notably, within PVN, some of these neurons co-expressed oxytocin (OT), c-Fos, and PRV. In summary, this study preliminarily proposed a potential uterus-brain neural circuit and suggests that olfactory cues may influence uterine activity via activation of circuit-specific neurons linked to uterus, including oxytocinergic populations. These findings provide experimental insight into the neural mechanisms underlying odor-mediated modulation of visceral function.

## Highlights

• Uterus-to-CNS multisynaptic pathway mapped via retrograde PRV tracing.
• Medial amygdala identified as a key olfactory relay for uterine modulation.
• Butyric acid activates oxytocin neurons in PVN linked transsynaptically to uterus.

## 1 Introduction

The uterus serves as the anatomical site for growth and development of both human and animal fetuses. Normal uterine contractions play a pivotal role in facilitating natural delivery and promoting uterine rejuvenation. Conversely, aberrant uterine contractions are intricately associated with adverse outcomes such as preterm birth, miscarriage, abnormal labor, postpartum hemorrhage, and non-pregnancy-related dysmenorrhea [1,2]. The CNS regulates the initiation, maintenance, and cessation of uterine contractions through a dual-regulatory system that integrates neuroendocrine and autonomic pathways [3,4]. A quintessential example of neuroendocrine control is provided by the OT neurons in PVN. These neurons release OT into the systemic circulation; this hormone acts directly on uterine smooth muscle to initiate and sustain powerful contractions, thereby serving as the primary effector of parturition [5]. Complementing this hormonal mechanism, the autonomic nervous system fine-tunes uterine activity via direct neural innervation [[6], [7], [8]]. Sympathetic inputs from the thoracolumbar spinal cord, for instance, can modulate myometrial activity with either excitatory or inhibitory effects, while afferent fibers concurrently relay crucial sensory information from uterus back to CNS [9].

Using pseudorabies virus (PRV) *trans*-synaptic tracing, previous studies have systematically mapped the multisynaptic neural circuits connecting uterus to CNS in rats [10,11]. These pathways, which span spinal cord, brainstem, and hypothalamus, are involved in the autonomic regulation of uterine function, mating-related behaviors, and estrogen-dependent neuromodulation, providing a foundational framework for understanding uterine innervation [10,11]. Although these two studies have detailed the anatomical connections between uterus and CNS in rats, they do not investigate the potential mechanisms by which the underlying neural circuits may influence uterine activity. Previous study has reported that when chamomile oil aromatherapy is administered during labor, while recording the duration, frequency, and intensity of uterine contractions as well as postpartum satisfaction, it is found that aromatherapy with chamomile oil reduces the intensity of uterine contractions and improved postpartum satisfaction when cervical dilation reached 5–7 cm [12]. Olfaction plays a significant role in modulating diverse physiological states, including emotion, stress, and reproductive function [13,14]. This modulation underlies clinical and behavioral observations, such as the effect of chamomile scent on uterine contractions in pregnant women [12], and the potential association between partner odor and unexplained pregnancy loss [15]. However, the underlying neural circuits and mechanisms through which odors may influence uterine activity remain unclear.

Given the limited availability of transgenic rats, it is challenging to perform functional validation manipulations in rats, such as optogenetics and chemogenetics. In contrast, transgenic mice have become the primary animal model for scientific research, particularly in neuroscience. Based on this, we employed PRV tracing, cholera toxin subunit B (CTB) tracing, odor exposure, and immunohistochemical staining to investigate the anatomical connections between uterus and CNS in mice, as well as the potential neural nodes through which odors may regulate uterine activity. We identified the anatomical connections between uterus and CNS in mice; however, no PRV-labeled neurons were observed in OB. Using CTB tracing, we established an anatomical link between OB and MeA. Through odor exposure and immunohistochemical staining, we found that BA activates neurons in PVN, CeA, and MeA, some of which are anatomically connected to uterus. Additionally, among the activated neurons in PVN that are connected to uterus, a subset was identified as oxytocinergic neurons. Our study provides an anatomical basis for the functional investigation of uterine-associated neural circuits in mice and provides preliminary elucidation of the neural mechanisms underlying the influence of odors on uterine activity.

## 2 Methods

### 2.1 Animals

All animal care and experimental procedures were approved by the Institutional Animal Care and Use Committee (IACUC) of The First Affiliated Hospital of Nanchang University (Approval No. CDYFY-IACUC-202302QR019) and performed in accordance with the Jiangxi Provincial Animal Care and Use Committee. We purchased female C57BL/6 mice for experiments and breeding from GemPharmatech LLC. (Nanjing, China). Mice aged 8–10 weeks and weighing 20–24 g were used throughout the study. For all experiments, mice were randomly assigned to experimental and control groups. The numbers of mice used for each experiment were listed in figure legends. Mice were housed in the animal facility of the First Affiliated Hospital of Nanchang University mouse barrier facility on a 12:12-h light-dark cycle with monitored temperature (21–23 °C) and humidity (30%–40%). Water and standard rodent chow were available ad libitum.

### 2.2 PRV virus injections

After female mice were anesthetized with a mixture of Tiletamine and Zolazepam (1:1 mixture, (25 mg + 25 mg)/Kg, intramuscularly into the hind limb), the uterus was exposed for injection under direct vision. 2 μL PRV-CAG-EGFP (Pseudorabies virus with CAG promoter driving EGFP expression, ≥2.00E+09 PFU/mL, BrainVTA, Wuhan, China) were injected into the left myometrium, made at a depth of 0.1–0.2 mm using a 30-gauge needle connected to a Hamilton syringe (10 μL) with a micromanipulator (ZS-CRO-R, Zhongshi Technology, Beijing, China) [16,17]. After each injection, the needle was kept in situ for 5 min, any efflux from the injection site to the uterus surface was immediately absorbed using cotton wool soaked with disinfectant and then the uterus subsequently wraped with gauzes to minimize nonspecific viral spread. After PRV injection, the uterus was put back into the abdominal cavity and the abdominal wound was sutured. After injection with PRV-CAG-EGFP, they were housed individually. The spinal cord and brain were sectioned and imaged 3–7 days later.

### 2.3 Imaging and analysis for PRV injections

On days 3, 5, and 7 following PRV injection, mice were deeply anesthetized and subsequently perfused with phosphate-buffered saline (PBS, 0.01 M), followed by a PBS solution containing 4% paraformaldehyde (w/v). The uterus, spinal cord, brain tissues were carefully dissected, removed, and post-fixed overnight at 4 °C. After fixation, uterine tissue was sectioned sagittally at a thickness of 30 μm using a cryostat (Leica CM1950). A series of sections were collected and stained with DAPI (5 μg/mL Roche, Germany) to verify the injection sites. For the spinal cord and brain tissues were embedded in 3% agarose and sectioned into 50 μm thick slices using a vibrating microtome (Leica VT1000S), with one slice collected every three serial sections. Both spinal cord and brain sections were incubated with DAPI. Images for all slices were acquired using a Leica Stellaris 5 confocal microscope (Germany) or Fluorescence slide microscope (PanoBrain, Meca Scientific, Tinyphoton Wuhan Technology Co., Ltd.). We used a 10× and 20× Plan Apochromat air objectives, and two laser wavelengths (405 nm, 488 nm). Image acquisition was controlled by Leica X software (Leica Stellaris 5, Germany) and Panolyzer software (Tinyphoton, Wuhan China). For analysis, target regions were manually delineated with reference to mouse brain atlas of Paxinos and Franklin (2nd edition) and atlas of the mouse spinal cord of Watson ao (2009). Spinal cord and brain were registered to a reference atlas. PRV-positive cells were then counted bilaterally in each region using ImageJ. The total number of infected cells per region was divided by the number of sections analyzed for that region to obtain the average count per section. Although Abercrombie correction was not applied, the systematic sampling of every third 50-μm-thick section minimized potential split-cell error.

### 2.4 Stereotaxic injections

Mice were maintained under anesthesia using 2% isoflurane delivered in pure oxygen and placed in a stereotaxic instrument (RWD, Shenzhen, China). Erythromycin eye ointment was applied to prevent corneal drying and a heat pad (RWD, Shenzhen, China) was used to hold body temperature at 37 °C. The injection was performed using the following stereotaxic coordinates for the MeA: −1.1 mm from bregma, 2.1 mm lateral from the midline, 5.0 mm vertical from the cortical surface; for CeA: −1.3 mm from bregma, 2.8 mm lateral from the midline, 4.0 mm vertical from the cortical surface. A small craniotomy hole was made using a dental drill (RWD, Shenzhen, China). 0.5 μL Alexa Fluor 555-conjugated cholera toxin subunit B (CTB555, 1.0 mg/mL Invitrogen, USA) was stereotaxically injected into the C57BL/6 female mice using a glass micropipette connected to a nanoliter injection pump (RWD, Shenzhen, China) and at a slow flow rate of 30 nl/min. The glass micropipette was left in place for 10 min before being slowly removed. Finally, the wound was sutured, topical antibiotics (neomycin) were applied to the surgical wound and analgesic (ketoprofen, 5 mg/kg) was injected subcutaneously. The animals were allowed to recover from anesthesia under a heat lamp.

### 2.5 Imaging and analysis for CTB injections

Three days after CTB injection, the mice underwent cardiac perfusion with PBS and 4% PFA. The brain tissue was carefully dissected out and then post-fixed overnight at 4 °C in 4% PFA. The brain tissue was embedded in 3% agarose and sectioned into 50 μm-thick slices using a vibratome (Leica VT1000S), with one slice collected every three serial sections. A series of sections were collected and incubated with DAPI to verify the injection sites. Only mice with verified injection sites were used for analysis. Histological verification showed that 3/6 mice in the MeA group and 3/7 mice in the CeA group had accurate injections and were included in the final analysis. We did not quantify the number of CTB-labeled neurons in the AOB and OB regions, but only examined these areas for the presence of CTB-positive neurons. Images of brain sections were acquired with a confocal microscope (Leica, Stellar is 5) or brain slice analyzer (meca scientific, PanoBrain).

### 2.6 Development of animal models for olfactory dysfunction

Adult female mice were anesthetized with Tiletamine and Zolazepam, then received bilateral intranasal administration of 0.7% Triton X-100 (100 μL in 0.01 M PBS) or equal volumes of PBS via a blunt-tipped microsyringe at a constant rate of 100 μL per minute [18]. Foraging behavior was assessed starting 2 days after 0.7% Triton X-100 treatment. Before testing, mice were acclimated to the experimental chamber for 30 min daily over 3 days. The testing arena was filled with a 3 cm layer of bedding, beneath which approximately 0.5 g of food pellets were hidden 0.5 cm below the surface at randomized locations. The latency to locate and retrieve the pellets using the forelimbs or mouth was recorded from the time the mouse was placed in the chamber until successful retrieval [19]. Each mouse was tested three times, and the average retrieval latency was calculated. If a mouse failed to find the food within the 300 s cut-off, it was gently removed and assigned the maximum time (300 s). Between trials, bedding was replaced to eliminate olfactory cues.

### 2.7 Odor exposure

The experimental animals were exposed to BA (Rhawn, Shanghai, China), a pungent yet non-predatory reference odorant. The study comprised three groups: intact mice (without any pretreatment) exposed to physiological saline; control mice (receiving intranasal PBS) exposed to BA; and mice with nasal mucosal injury (induced by intranasal administration of 0.7% Triton X-100) exposed to BA. Each substance was applied to sterile gauze. To maintain consistency in odor intensity, volume standards previously defined were followed [20]. Specifically, 105 μL of BA or an equivalent volume of saline was applied to a sterile gauze pad measuring 5 cm × 5 cm, which was then positioned within a transparent enclosure measuring 17 cm × 28 cm × 12 cm. Each mouse underwent a single 15 min session in the enclosure with either saline or BA exposure. Following the odor exposure, all mice were transferred to a clean environment. After 90 min, the mice were perfused.

### 2.8 Immunostaining

Mice with nasal mucosal injury and control mice were exposed to odor or saline for 15 min two days after the procedure. Perfusion and post-fixation of tissues were performed 90 min after removal of the odor source. Some experimental mice were pre-injected with PRV into the uterus, followed by odor exposure and subsequent experiments 7 days later. Agarose-embedded brain tissues were coronally sectioned into 30-μm-thick slices using a vibratome, one of every two consecutive sections was collected staining. After rinsing with PBS, the brain slices were blocked with 0.3% Triton X-100 and a blocking buffer (P0260, Beyotime, Shanghai, China). For odor exposure experiments without PRV injection, sections containing the PVN were incubated with OT antibody (1:300, rabbit, Invitrogen, PA5-112637, USA) and c-Fos antibody (1:2000, rat, Synaptic System, 226017, Germany), while sections containing the CeA, MeA, and OB were incubated only with c-Fos antibody. For brain slices from mice subjected to PRV injection followed by odor exposure, sections containing the PVN were incubated with OT antibody and c-Fos antibody. After primary antibody incubation for 48 h, the slices were washed three times with PBS for 10 min each, followed by incubation with corresponding secondary antibodies (1:1000, goat anti-rat IgG conjugated with Alexa Fluor 647, Invitrogen, A-21247, USA and donkey anti-rabbit IgG conjugated with Alexa Fluor 594, Invitrogen, A-21207, USA) for 2 h. After secondary antibody incubation, the slices were incubated with DAPI for 10 min and washed three times with PBS for 10 min each.

Finally, the slices were mounted, coverslipped, and imaged using a confocal microscope (Leica, Stellaris 5) or a brain slice analyzer (meca scientific, PanoBrain). We used a 10× Plan Apochromat air objectives, and four laser wavelengths (405 nm, 488 nm, 561 nm, 635 nm). Image acquisition was controlled by Leica X software (Leica Stellaris 5, Germany) and Panolyzer software (Tinyphoton, Wuhan China). Target regions were manually outlined according to the referenced brain atlas. For each animal, 6–8 sections per region were analyzed. For the analysis of c-Fos-positive neurons, we quantified the number and the area occupied by these neurons in bilateral regions of interest (ROIs) using ImageJ. Neuronal density was then calculated as the number of cells divided by the measured area. For co-labeled neurons (PRV with c-Fos and/or OT), the number of co-labeled neurons in the target brain regions was quantified using ImageJ. These counts were then corrected for potential split-cell error using Abercrombie's method, and the corrected values were subsequently normalized by the total number of tissue sections analyzed.

### 2.9 Quantification and statistical analysis

Data was plotted as mean ± SEM in bar graphs and error bars. Cells counting was conducted by an experimenter blinded to the experimental conditions. Data were analyzed using GraphPad Prism (v8.0, GraphPad Software). Non-parametric alternatives (Mann-Whitney tests) were applied when data violated normality assumptions. The p-values were calculated by non-paired test, paired test and two-way ANOVA followed by Tukey's post hoc test. Statistical Significance level was set at non-significance (ns), ∗p < 0.05, ∗∗p < 0.01, ∗∗∗p < 0.001.

## 3 Result

### 3.1 Pseudorabies virus infection in the CNS

**Fig. 1:**
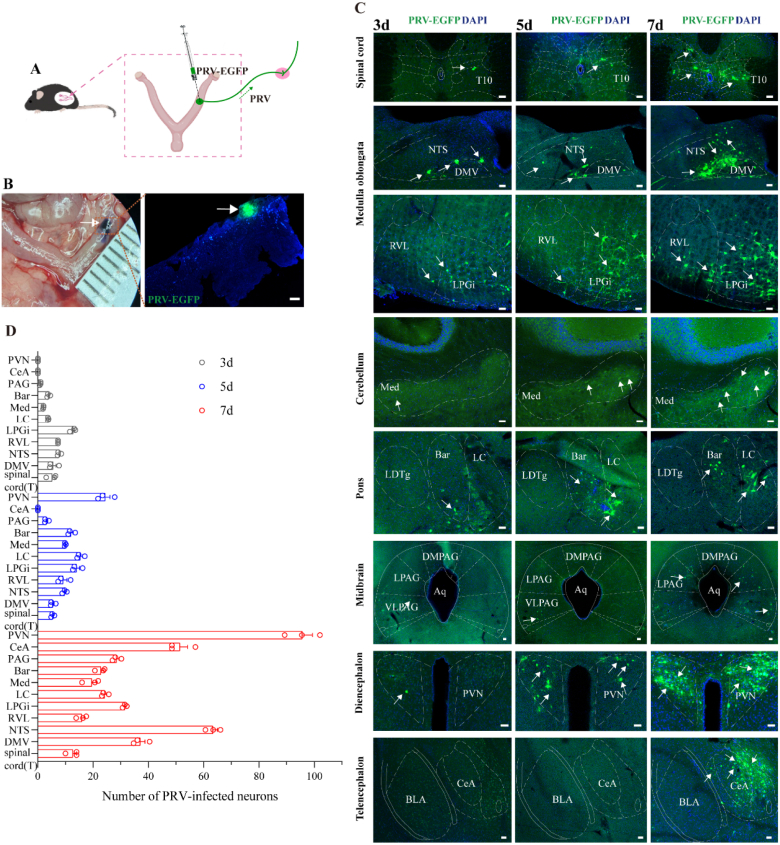
Schematic distribution of PRV-infected cells in the CNS at different time points following PRV infection in the myometrium of uterus. (A) Schematic of PRV-EGFP injection into the uterine myometrium. (B) Representative diagram of the actual injection site into the uterine myometrium (left), representative diagram of the PRV-EGFP infection site in the myometrium of uterus (right). Scale bar, 200 μm (C) Confocal images show the distribution of PRV-infected neurons in CNS at different time points post-injection. Scale bar, 50 μm. White arrow, PRV-positive neurons. (D) Histogram showing the number of PRV-infected neurons in the CNS (n = 3). T10, thoracic spinal cord 10; NTS, solitary tract nucleus; DMV, dorsal motor nucleus of vagus; RVL, rostral ventrolateral reticular nucleus; Med, medial (fastigial) cerebellar nucleus; LPGi, lateral paragigant cell nucleus; LDTg, laterodorsal tegmental nucleus; Bar, barrington's nucleus; LC, locus coeruleus nucleus; Aq, aqueduct; PAG, periventricular gray matter; DMPAG, dorsomedial periaqueductal gray; LPAG, lateral periaqueductal gray; VLPAG, ventrolateral periaqueductal gray; PVN, paraventricular thalamic nucleus; BLA, basolateral amygdaloid nucleus; CeA, basolateral amygdaloid nucleus.

To investigate the neuroanatomical connections between the uterus and the CNS in mice, PRV-CAG-EGFP was injected into the myometrium of the left uterine horn of mice for 3 d, 5 d or 7 d (Fig. 1A). Since the injection was visible, sagittal section imaging of the uterus revealed that PRV primarily infected the myometrium (Fig. 1B). The extent and quantity of PRV infection in the CNS exhibited time-dependent characteristics. At 3 days post-inoculation, viral labeling was largely confined to the spinal cord and medulla oblongata (Fig. 1C). By day 5, PRV-labeled neurons were densely present in several nuclei involved in autonomic and monoaminergic regulation, such as the rostral ventrolateral medulla (RVL) and the lateral paragigantocellular nucleus (LPGi) (Fig. 1C). After 7 days, the distribution of labeled neurons was the most extensive, extending into multiple forebrain regions including CeA and PVN (Fig. 1C). Quantitative analysis of labeled neuron counts across these time points is summarized in Fig. 1D. A detailed breakdown of the specific distribution patterns is provided in the following section.

The following section focuses on analyzing PRV-infected neurons in the CNS at 7 days post-injection. We examined the distribution of PRV infection by segmenting the central nervous system into several regions based on their proximity to the uterus, using spinal cord and brain stereotaxic atlases as references. The specific distribution overviews were as follows.

#### 3.1.1 Spinal cord

**Fig. 2:**
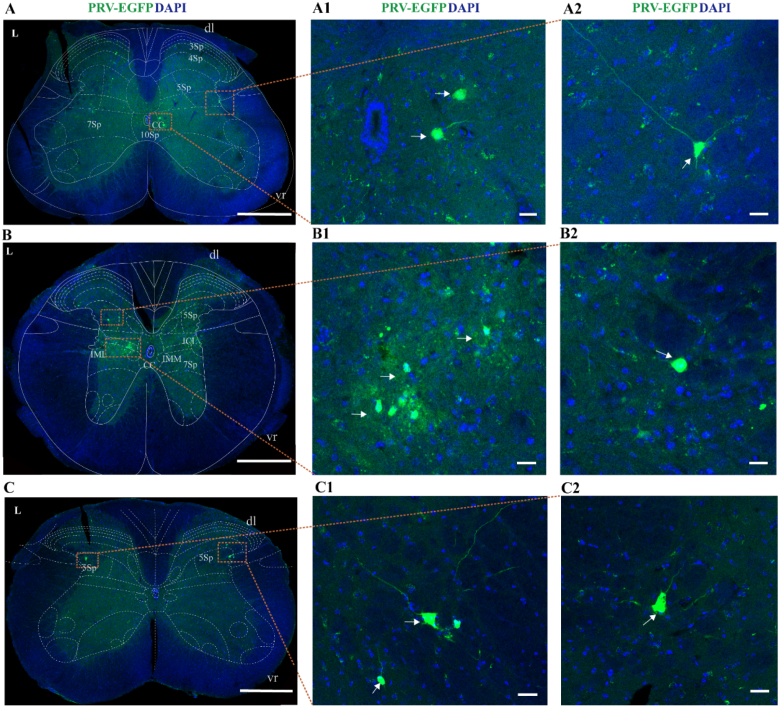
Overview of PRV-infected cell distribution in the spinal cord 7 days post-PRV injection. (A) Sparsely scattered PRV-infected neurons were observed in the lumbosacral spinal cord (L6 segment). L, left side. Scale bar, 500 μm. Panels A1 and A2 show magnified views of the PRV-infected area in figure A. Scale bar, 20 μm (B) A small number of PRV-infected neurons were distributed in the thoracic spinal cord (T10 segment). L, left side. Scale bar, 500 μm. Panels B1 and B2 show magnified views of the PRV-infected area in figure B. Scale bar, 20 μm (C) A few PRV-infected neurons were distributed in the cervical spinal cord (C6 segment). L, left side. Scale bar, 500 μm. Panels C1 and C2 show magnified views of the PRV-infected area in figure C. Scale bar, 20 μm. 10Sp, lamina X; 7Sp, lamina Ⅶ; 5Sp, lamina Ⅴ; dl, dorsolat fasc; vr, ventral root; CC, central canal; IML, intermediolateral nucleus; IMM, intermediomedial nucleus; ICl, intercalated nucleus; 7Sp, lamina Ⅶ; 5Sp, lamina Ⅴ; dl, dorsolat fasc; vr, ventral root; 5Sp, lamina Ⅴ; dl, dorsolat fasc; vr, ventral root; L, left side.

In the lumbosacral spinal cord (L6–S1), PRV-infected cells were primarily distributed in autonomic and sensory relay nuclei, in the lamina Ⅴ, lamina Ⅶ and lamina X (surrounding the central canal) (Fig. 2A). In the thoracic spinal cord (T10–T13), PRV-infected neurons were primarily located in the intermediolateral nucleus (IML, Rexed lamina Ⅶ), intercalated nucleus (ICl), lamina X and intermediomedial nucleus (IMM) (Fig. 2B). In the cervical spinal cord, PRV-infected cells were sparsely scattered in the lamina Ⅴ and near the dorsal horn (Fig. 2C). PRV-infected neurons were observed in the bilaterally aforementioned regions, indicating the existence of bilateral-innervation within neural pathways.

#### 3.1.2 Medulla oblongata (bregma −8.20 to −7.30 mm)

**Fig. 3:**
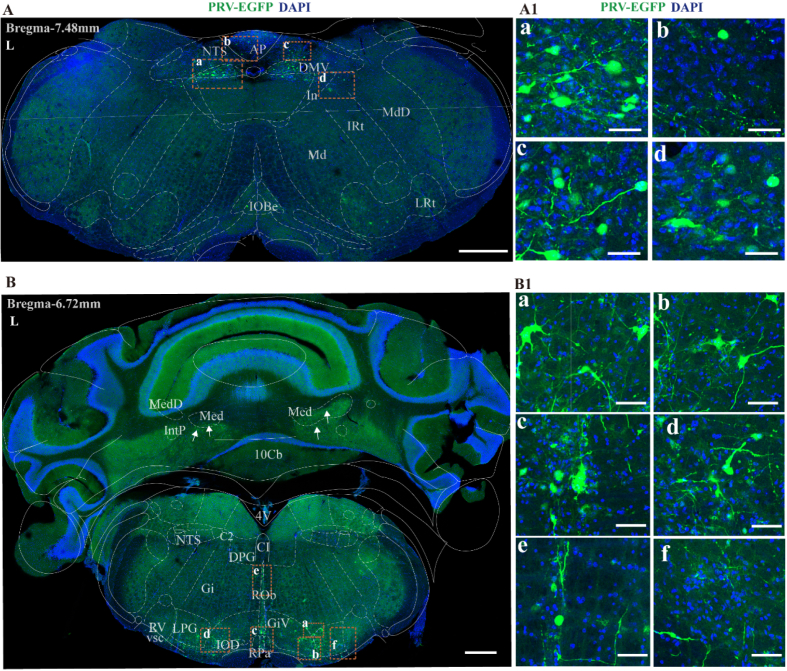
Overview of PRV-infected cell distribution in the medulla oblongata 7 days post-PRV injection. (A) Dense PRV-infected neurons were distributed in the nucleus of the solitary tract, scale bar 500 μm. Panels A1a to A1d show magnified views of selected PRV-infected areas from figure A, scale bar 50 μm (B) Representative figure showing the distribution of PRV infection from rostral to middle segment of the caudal medulla (Bregma −6.72 mm), scale bar 500 μm. Panels B1a to B1f show magnified views of selected PRV-infected areas from Figure B, scale bar 50 μm. DMV, dorsal motor nucleus of vagus; NTS, solitary tract nucleus; AP, area postrema; Gi, gigantocellular reticular nucleus; LPGi, lateral paragigantocellular; GiV, gigantocellular reticular nucleus, ventral; RPa, raphe pallidus nucleus; IOD, inferior olive, dorsal nucleus; Rob, raphe obscurus nucleus; RVL, rostral ventrolateral reticular nucleus; vsc, ventral spinocerebellar tract; Gi, reticular nucleus; C2, C2 cell group. Med, medial (fastigial) cerebellar nucleus; 10Cb, 10th Cerebellar lobule; L, left side.

For clarity of description, we divided the medulla oblongata into anterior and posterior parts according to the brain atlas. The posterior part spans from bregma −8.20 mm to −7.30 mm, and the anterior part from bregma −7.30 mm to −6.60 mm. In the posterior part of medulla oblongata, PRV-infected neurons were prominently observed in the dorsal motor vagal nucleus (DMV), the nucleus tractus solitarius (NTS) and the area postrema (AP) (Fig. 3A). In the anterior part of medulla oblongata, PRV-infected neurons were observed in the lateral paragigantocellular nucleus (LPGi), ventral part of the gigantocellular reticular nucleus (GiV), raphe obscurus nucleus (Rob), raphe pallidus nucleus (RPa), rostroventrolateral reticular nucleus (RVL), inferior olive of the dorsal nucleus (IOD), ventral spinocerebellar tract (vsc) and reticular nucleus (Gi) (Fig. 3B). In addition, a small number of infected neurons were observed in both the medial and paracellular populations of the NTS, cerebellar nucleus, as well as in the C2 cell population (Fig. 3B).

Lower levels of infection were found in the paramedian reticular nucleus (PMn), intermediate reticular nucleus (IRt), lateral reticular nucleus (LRt), alpha part (GiA), alpha part of the parvocellular reticular nucleus (PCRtA), oral part of the spinal trigeminal nucleus (Sp5O), spinal vestibular nucleus (SpVe), linear nucleus (Li), dorsal paragigantocellular nucleus (DPGi), raphe magnus nucleus (RMg) (data not shown).

#### 3.1.3 Pons (bregma −6.70 to −5.00 mm)

**Fig. 4:**
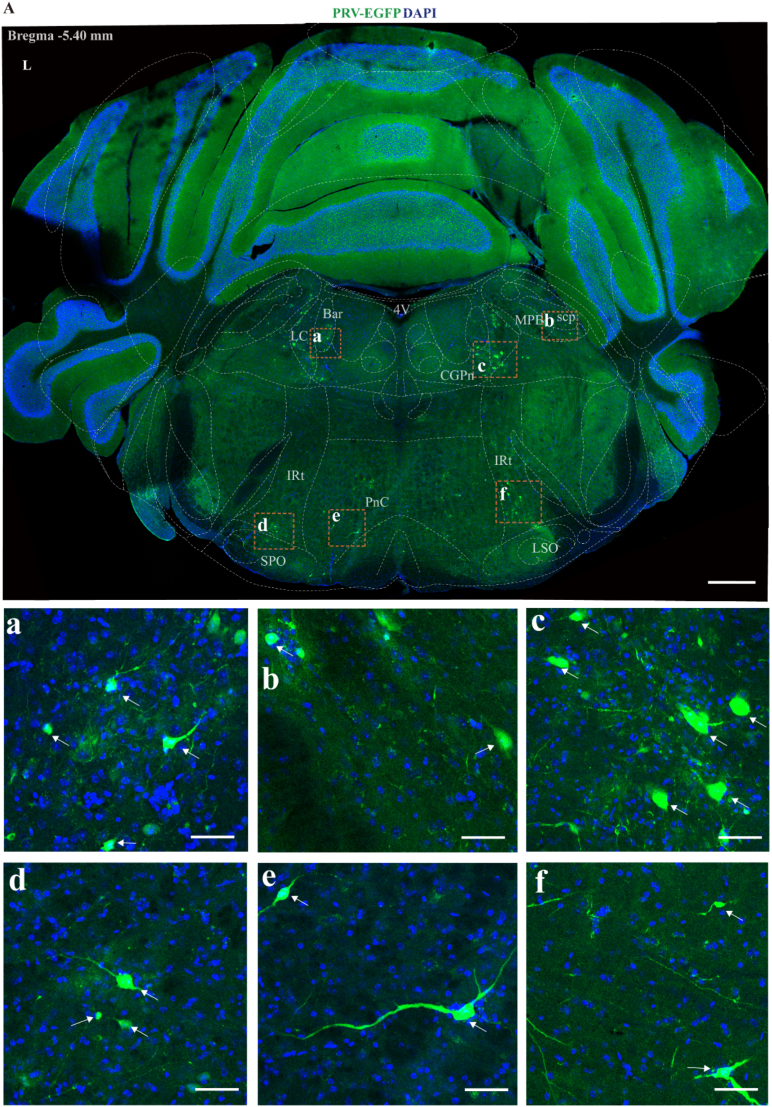
Overview of PRV-infected cell distribution in the pons 7 days post-PRV injection. (A) (Upper part of Figure A) Representative figure showing the distribution of PRV infection in pons area (Bregma −5.40 mm), scale bar 500 μm. (Lower panel of Figure A) Panels a to f show magnified views of selected PRV-infected areas from upper part of Figure A, scale bar 50 μm. 4V, 4th ventricle; Bar, Barrington's nucleus; LC, locus coeruleus; MPB, medial parabrachial nucleus; scp, superior cerebellar peduncle; CGPn, central gray of the pons; SPO, superior paraolivary nucleus; PnC, pontine reticular nucleus, caudal part; IRt, intermediate reticular nucleus; LSO, lateral superior olive. L, left side.

In the pons, PRV-infected neurons were distributed in the barrington's nucleus (Bar), locus coeruleus (LC), medial parabrachial nucleus (MPB), superior cerebellar peduncle (scp), central gray of the pons (CGPn), intermediate reticular nucleus (IRt), superior paraolivary nucleus (SPO), pontine reticular nucleus (PnC) (Fig. 4).

#### 3.1.4 Midbrain (bregma −5.00 to −3.00 mm)

**Fig. 5:**
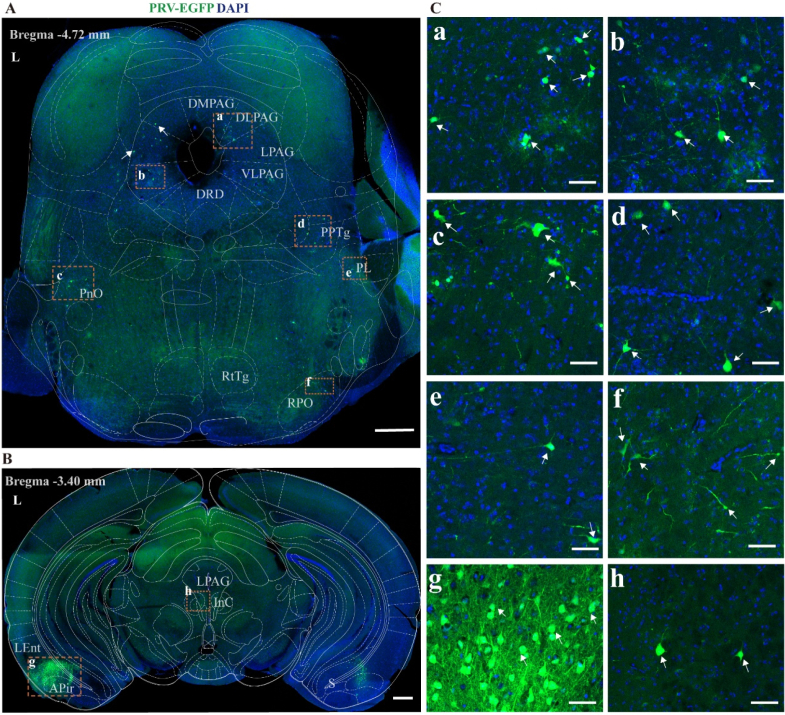
Overview of PRV-infected cell distribution in the midbrain at 7 days post-injection. (A) Representative figure showing the distribution of PRV infection in posterior parts of midbrain gray matter section (Bregma −4.72 mm), scale bar 500 μm (B) Representative figure showing the distribution of PRV infection in anterior parts of midbrain section (Bregma −3.4 mm), scale bar 500 μm (C) Panels a to f show magnified views of selected PRV-infected areas from Figure A, B, scale bar 50 μm. DMPAG, dorsomedial periaqueductal gray; DLPAG, dorsolateral periaqueductal gray; LPAG, lateral periaqueductal gray; VLPAG, ventrolateral periaqueductal gray; DRD, dorsal raphe nucleus, dorsal part; PPTg, pedunculopontine tegmental nucleus; RPO, rostral periolivary region; PnO, pontine reticular nucleus, oral part; PL, paralemniscal nucleus; RtTg, reticulotegmental nucleus of the pons; LEnt, lateral entorhinal cortex; APir, amygdalopiriform area; S, subiculum; InC, interstitial nucleus of Cajal. L, left side.

At the posterior parts of midbrain gray matter, PRV-infected neurons were sparsely distributed in the lateral periaqueductal gray (LPAG), dorsolateral periaqueductal gray (DLPAG), ventrolateral periaqueductal gray (VLPAG), dorsomedial periaqueductal gray (DMPAG), pedunculopontine tegmental nucleus (PPTg), rostral periolivary region (RPO), pontine reticular nucleus, oral part (PnO) and paralemniscal nucleus (PL) (Fig. 5A).

At the anterior parts of midbrain, to our surprise, we observed a dense cluster of PRV-infected neurons in the amygdalopiriform area (APir) and lateral entorhinal cortex (LEnt), with a notably higher density on the left side compared with the right (Fig. 5B). This suggests that in the APir and LEnt, although the innervation is bilateral, the ipsilateral projection appears to be predominant.

#### 3.1.5 Hypothalamus (bregma −3.00 to 0.00 mm)

**Fig. 6:**
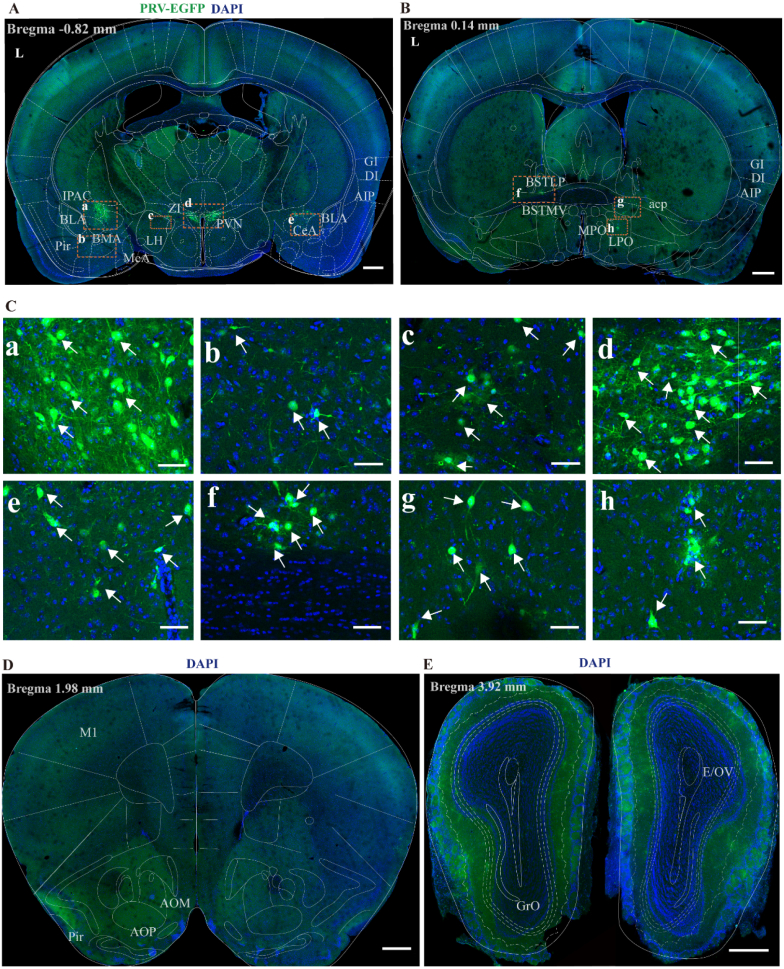
Overview of PRV-infected cell distribution in prosencephalon at 7 days post-injection. (A) Representative figure showing the distribution of PRV infection in hypothalamus section (Bregma −0.82 mm), scale bar 500 μm (B) Representative figure showing the distribution of PRV infection in telencephalon section (Bregma 0.14 mm), scale bar 500 μm (C) Panels a to h show magnified views of selected PRV-infected areas from Figure A, B, scale bar 50 μm. (D) Representative figure showing the distribution of non-PRV infection in frontal cortex section (Bregma 1.98 mm), scale bar 500 μm (E) Representative figure showing the distribution of non-PRV infection in olfactory bulb section (Bregma 3.92 mm), scale bar 500 μm. BLA, basolateral amygdala; CeA, central amygdaloid nucleus; LH, lateral hypothalamic area; PVN, paraventricular hypothalamic nucleus; Pir, piriform cortex; MeA, medial amygdala; GI, granular insular cortex; DI, dysgranular insular cortex; AIP, posterior part of agranular insular cortex; acp, posterior anterior commissure; BSTMV, medial ventral part of the bed of the striatum; LPO, lateral preoptic area; MPO, medial preoptic nucleus; BSTLP, posterior lateral part of the bed of the striatum; AOP, anterior olfactory nucleus, posterior part; AOM, anterior olfactory nucleus, medial part; GrO, granular cell layer of the olfactory bulb; E/OV, ependymal and subendymal layer/olfactory ventricle; L, left side.

In hypothalamus, PRV-infected neurons were distributed across several regions, including the amygdala complex, agranular insular cortex (AIP), piriform cortex (Pir), anterior commissure hind limb (IPAC), granular insular cortex (GI), semi-granular insular cortex (DI), posterior anterior commissure (acp), medial ventral part of the bed of the striatum (BSTMV), and posterior lateral part of the bed of the striatum (BSTLP), the paraventricular nucleus (PVN), medial preoptic area (MPO), lateral preoptic area (LPO), lateral hypothalamus (LH) and zona incerta (ZI) (Fig. 6A–C). PRV-infected neurons were also found in the ventromedial hypothalamus (VMH) (data not shown).

Among these brain regions, the IPAC and PVN contained a higher number of PRV-infected neurons compared with other regions. Furthermore, the IPAC also exhibited a predominance of ipsilateral innervation. Additionally, within the amygdalar complex, a small number of PRV-infected neurons were distributed in the basolateral amygdala (BLA), medial basal amygdala (BMA), CeA, and MeA (Fig. 6A).

#### 3.1.6 Additional sites (bregma 3.00 to 5.00 mm)

In the anastomosing region of the telencephalon and the olfactory bulb, no infected neurons were found (Fig. 6D and E).

Together, these data reveal transsynaptic connections between the uterus and CNS in mice.

### 3.2 The olfactory bulb is anatomically connected to the MeA not the CeA

**Fig. 7:**
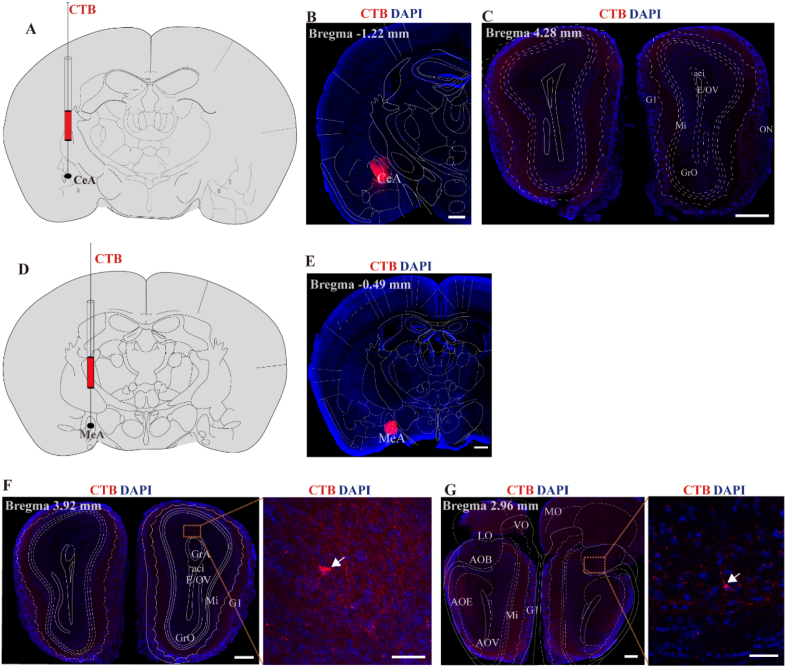
Projective neurons were observed in the OB and AOB following the CTB injected into the MeA. (A and D) Schematic diagram of CTB injection into the CeA and MeA. (B and E) Representative image of CTB555 injection site in the CeA and MeA, scale bar, 500 μm (C) Representative image shows no CTB555-labeled neurons in the OB, scale bar, 500 μm (F and G) Representative image shows a few CTB555-labeled neurons in the OB (F) and AOB (G) after CTB injections into MeA. Scale bar, 500 μm. The right panel shows a magnified view. Scale bar, 50 μm.

Previous clinical study has found that exposure to chamomile odor can lead to alterations in uterine activity [12], yet the underlying neural circuit mechanisms remain unclear. In the OB or accessory olfactory bulb (AOB) (which serve as odor-processing centers), our uterine PRV tracing data revealed no PRV-infected cells. Therefore, the question arises: how does odor information ultimately influence uterine activity after being processed by the OB? Previous study has reported that odors may modulate uterine activity by affecting emotional regulatory nodes [12,15]. The amygdala functions as an emotional center, CeA is involved in the integration of emotional and stress responses [21,22], while MeA serves as a critical hub for processing olfactory social signals, directly receiving inputs from the OB and integrating pheromonal signals with social environmental information [22,23]. MeA and CeA are functionally interconnected [22,24,25], participating in the integration of emotional and stress responses [22]. Furthermore, this study found that PRV-infected neurons in MeA and CeA. Therefore, to further investigate whether odor information might be relayed through the CeA and MeA, we first injected the retrograde tracer CTB into the CeA and MeA to map their anatomical connections with the OB or AOB (Fig. 7A, B, D, E). In mice injected with CTB into the CeA, no CTB-positive neurons were observed in the OB or AOB (Fig. 7C). However, in mice injected with CTB into the MeA, CTB-positive neurons were found in both the OB and AOB, and these neurons were distributed in the GrO layer (Fig. 7F and G). Our data identify anatomical connections between the MeA and both the OB and AOB.

### 3.3 Butyric acid exposure activates specific brain regions that have anatomical connections to the uterus

To identify which odorants can activate potential neural nodes involved in odor-influenced uterine activity, such as the OB, PVN, MeA, and CeA, the mice were exposed to BA, musk, fox urine, or chamomile odor, followed by c-Fos staining. We observed that only BA significantly activated the OB, PVN, MeA, and CeA, whereas the other three odorants did not significantly activate these four brain regions (Supplementary Fig. 1). To investigate whether BA could alter uterine activity, we recorded uterine electromyographic (EMG) activity and administered either OT or BA. However, under anesthesia, neither OT nor BA changed uterine EMG activity (data not shown).

**Fig. 8:**
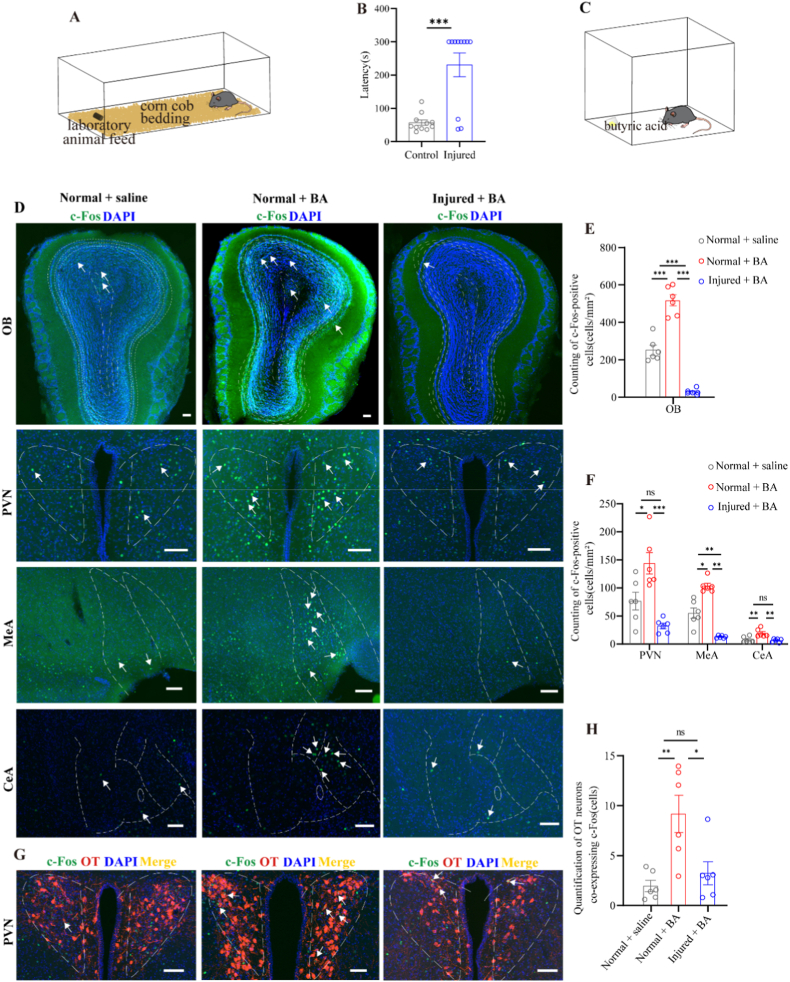
BA exposure activates neurons in certain brain regions anatomically connected to the uterus. (A) Schematic diagram of foraging behavior. (B) Histograms of foraging latency in mice (n = 11), ∗∗∗*p* < 0.001, unpaired *t*-test. (C) Schematic diagram of the BA odor exposure experiment. (D) Representative images of c-Fos staining in the OB, PVN, MeA, and CeA. (G and H) Representative images of c-Fos staining and OT staining in the PVN (G), Histograms showing the counts of c-Fos-positive and OT-positive neurons. (E and F) Histogram of c-Fos expression in the OB (E), PVN, MeA, and CeA (F). n = 6, *ns*, no significant; *∗p* < 0.05, ∗∗*p* < 0.01, *∗∗∗p* < 0.001, two-way ANOVA followed by Tukey's post hoc test.

To further verify that odor exposure activates downstream brain regions by first activating the OB, we established a nasal mucosal injury model using 0.7% Triton X-100 (which was found to prolong food-seeking latency) and then exposed the mice to BA followed by c-Fos staining (Fig. 8A–C). We found that OB lesioning suppressed the activation of the OB, PVN, MeA, and CeA induced by BA (Fig. 8D–F). Next, we performed c-Fos and OT co-staining in the PVN and found that BA exposure activated a subset of OT-positive neurons (Fig. 8 G). The number of these activated neurons was significantly greater than that in both the control group and the nasal mucosal injury group (Fig. 8H). These data suggest that odor exposure may influence uterine activity by activating brain regions that are anatomically connected to the uterus.

### 3.4 A subset of the neurons activated by butyric acid exposure are transsynaptically connected to the uterus

**Fig. 9:**
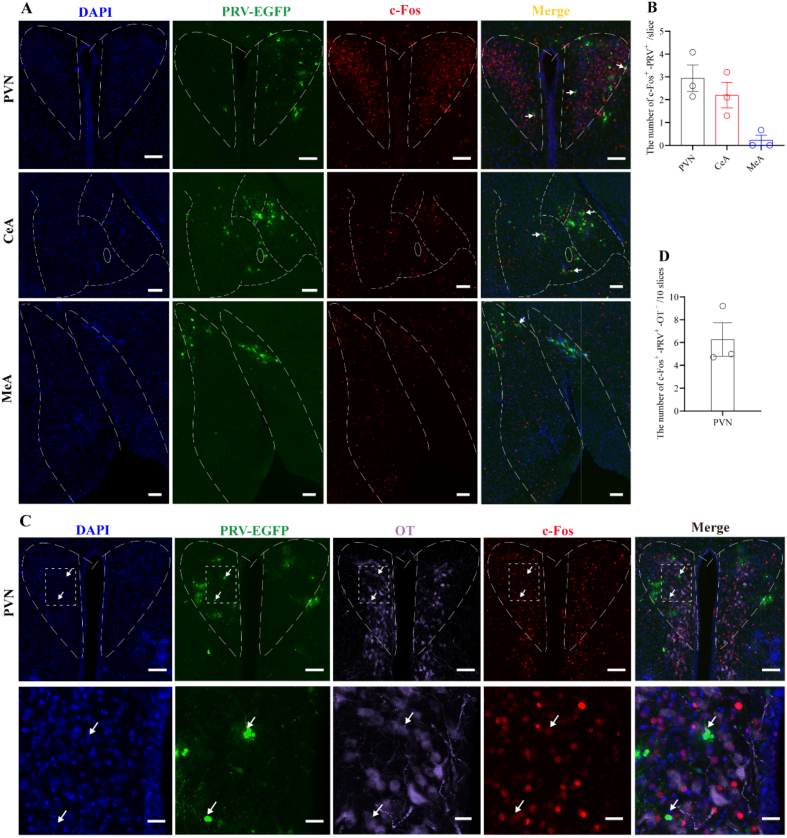
Exposure to BA odor activates neurons in some brain regions that are anatomically connected to the uterus. (A) Representative images of c-Fos immunostaining in the PVN, CeA, and MeA after PRV injection in mice, scale bar 100 μm (B) Histogram of PRV-positive and c-Fos-positive cells in the PVN, CeA, and MeA (n = 3). (C, D) Representative images of c-Fos and OT immunostaining in the PVN after PRV injection in mice (C), histogram of PRV-positive, c-Fos-positive and OT cells in the PVN (D, n = 3). The lower panel in C shows an enlarged view of the white region in the upper panel. White arrows indicate triple-labeled neurons, PRV-labeled OT neurons showed weak c-Fos labeling. Scale bars, 100 μm (upper panel) and 30 μm (lower panel).

To investigate whether neurons activated by BA exposure are transsynaptically connected to the uterus, we first injected PRV. Seven days later, mice were exposed to BA stimulation and subsequently processed for immunohistochemical staining. We observed neurons co-labeled for c-Fos and PRV in the PVN, CeA, and MeA (Fig. 9A and B). Furthermore, triple-labeled neurons positive for PRV, OT, and c-Fos were observed in the PVN (Fig. 9C and D). Notably, PRV-labeled OT neurons showed weak c-Fos immunoreactivity under this experimental condition. These data suggest that a subset of the neurons activated by BA exposure are transsynaptically connected to the uterus, and that within the PVN, a portion of these are oxytocinergic neurons.

## 4 Discussion

**Fig. 10:**
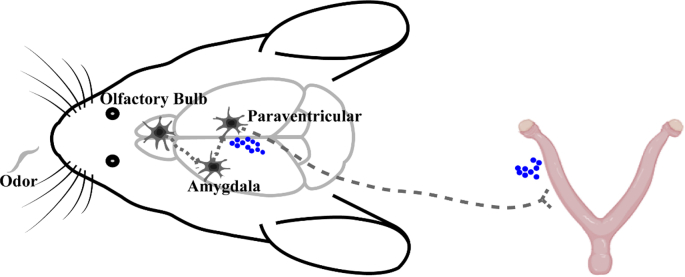
Proposed mechanism: after perceiving specific odors, the olfactory bulb transmits signals to amygdala and PVN. The PVN, in turn, may trigger the release of the neuroendocrine hormone oxytocin (blue circle) or send autonomic signals, thereby influencing uterine activity.

This study identifies a polysynaptic neural circuit linking the uterus of mice to the CNS and reveals a potential pathway through which, BA, may influence uterine activity by engaging this circuitry (Fig. 10).

Our PRV tracing results revealed a time-dependent, hierarchically organized pathway from the uterus to the CNS. In most brain regions, PRV-labeled neurons exhibited a bilateral distribution, with no marked quantitative disparity between the two sides. This pattern is likely attributable to the relatively low overall number of infected neurons in these areas. However, within the densely PRV-infected IPAC and piriform cortex, the number of infected neurons on the ipsilateral side (relative to the injection site) was significantly greater than that on the contralateral side. This suggests that uterine innervation encompasses both bilateral and ipsilateral projections, with certain brain regions exhibiting a predominance of ipsilateral neuronal innervation. The discovery of a dense cluster of PRV-labeled neurons in the IPAC, APir and LEnt was unexpected. This finding suggests that the olfactory-limbic system may be involved in the modulation of uterine activity by olfactory cues [[26], [27], [28], [29], [30]]. Some key autonomic and stress-response nodes, including the NTS, RVL, LC, and PVN, were heavily labeled, which may be related to their involvement in regulating visceral functions [31,32].

Previous studies by Papka et al. and Erskine et al. [10,11] have extensively mapped PRV-labeled neurons in the rat CNS. We systematically compared the results of this mouse study with rat PRV tracing data (Supplementary Table 1). The PRV injection site in this study differed from that in rat studies (unilateral uterine horn injection in this work vs. primarily cervical/uterine body injection in rats). However, the time-dependent progression of retrograde PRV transport and the key relay nuclei involved (e.g., IML, NTS, PAG, PVN) were highly consistent. Nevertheless, several notable differences were observed. First, suprachiasmatic nucleus (SCH) labeling clearly reported in rat studies was not prominently detected here, possibly because the SCH is located caudally and more prone to loss during vibratome sectioning and floating. Second, at late infection stages, more intense and distinctly ipsilateral viral labeling was observed in APir and LEnt in our study, a phenomenon that may be attributed to the higher expression efficiency of the PRV-CAG-EGFP viral vector used here. Finally, differences in relative labeling intensity were also noted; for instance, Barrington's nucleus, described as “extremely dense” in rat studies [10,11], was clearly labeled in our work but not to the same degree, which could be related to the difference in injection sites.

Building on the clinical observation that odors can alter uterine activity [12] and odors may modulate uterine activity by affecting emotional states [12,33]. The amygdala functions as an emotional center, in which the CeA integrates emotional and stress responses, while the MeA acts as a critical hub for processing olfactory social signals [[21], [22], [23], [24]]. Based on this, we sought to identify the polysynaptically connected nodes through which olfactory stimuli might modulate uterine activity. Previous anterograde tracing studies had shown that MeA projects to AOB [22,24]. Besides, other studies had confirmed that MeA receives direct inputs from both the OB and AOB [22,34], though it remains unclear whether the CeA receives similar direct olfactory afferents [22,34]. In this study, by injecting CTB into the MeA, we aimed to investigate whether the MeA receives inputs from the AOB. Our results show that the AOB projects to the MeA, which is consistent with previous studies [35]. On the other hand, Pardo-Bellver et al. found that MeA projects to the AOB [24], indicating the existence of reciprocal anatomical connections between the MeA and AOB [22,24,25]. Additionally, Pardo-Bellver et al. reported that the MeA projects to the CeA [24], which aligns with our finding from CTB retrograde tracing in the CeA (Supplementary Fig. 2). Together, evidence from these complementary tracing directions suggests that the MeA may share bidirectional anatomical connectivity with the olfactory system. In contrast, the CeA could integrate olfactory information indirectly through intermediate nodes, rather than directly receiving peripheral olfactory input. Although no direct polysynaptic connection was found between the uterus and the OB, our anatomical tracing revealed that the MeA receives input from both the main and accessory olfactory bulbs and is itself polysynaptically connected to the uterus. This positions the MeA as a critical node for olfactory information to access uterus control circuits.

Functional studies using c-Fos demonstrated that butyric acid activates key nodes within a potential circuitry, such as the OB, PVN, MeA, and CeA. This activation was contingent on an intact OB, as nasal mucosal lesioning abolished it. Further investigation revealed that a subset of the neurons activated by BA established polysynaptic connections with the uterus. Moreover, within the PVN, a portion of the activated neurons that were polysynaptically connected to the uterus were oxytocinergic neurons. Although variations in infection efficiency exist among different batches of PRV virus (Figs. 1C and 9C), which may have reduced the statistical power of some quantitative analyses, this technical variable has minimal impact on the conclusion of our study. This observation proposes the possibility that BA-responsive neurons, including oxytocin-expressing cells, reside within a hypothalamic nucleus that is anatomically linked to the uterus and is implicated in reproductive functions [[36], [37], [38]].

Collectively, these findings may support a model wherein BA activates the OB and subsequently the MeA. The MeA, in turn, may relay this signal to downstream limbic and hypothalamic regions, which are part of the efferent network governing the uterus. The activation of uterus-connected OT neurons in the PVN suggests a plausible neuroendocrine mechanism for odor-induced modulation. In addition, within the PVN, a subset of the BA-activated neurons that are polysynaptically connected to the uterus were not oxytocinergic. This suggests that autonomic mechanisms may also be involved, acting in concert with the neuroendocrine pathway.

This study has several limitations. The use of PRV, while excellent for mapping connections, does not distinguish between direct and indirect polysynaptic inputs to a given neuron. Part of the functional experiments were conducted under anesthesia, which may suppress certain neural or muscular responses. Another portion relied on c-Fos staining, which has relatively low temporal resolution and event correlation. Future studies should employ multi-channel electrophysiology or fiber photometry to record real-time neuronal responses in relevant brain regions during odor exposure in awake, behaving animals, while simultaneously monitoring uterine activity via implanted pressure transducers. Subsequent application of functional manipulation techniques (e.g., chemogenetics/optogenetics), combined with such physiological recordings, will be essential to investigate the causal role of the identified potential circuit in mediating odor-induced modulation of uterine function.

## 5 Conclusion

In summary, we have delineated a central neural network connected to the mouse uterus and identified butyric acid as an olfactory stimulus capable of activating a subset of neurons within this network. Our data suggest that the MeA may serve as a key node, relaying olfactory signals to established visceral control centers, such as the oxytocinergic neurons in the PVN, thereby potentially modulating uterine activity. This work provides a foundational neural circuit mechanism for understanding how environmental chemosensory signals can influence reproductive physiology.

## Ethics approval and consent to participate

This study protocol was approved by by the Jiangxi Provincial Animal Care and Use Committee and conducted in accordance with the guidelines of the Institutional Animal Care and Use Committee of the First Affiliated Hospital of Nanchang University.

Consent for publication (Approval No. CDYFY-IACUC-202302QR019).

## CRediT authorship contribution statement

**Hehua Wang:** Data curation, Formal analysis, Methodology, Validation, Writing – original draft. **Jinqiong Zhan:** Data curation, Investigation, Methodology. **Qingyue Cao:** Data curation, Investigation, Methodology. **Jingjing Hu:** Data curation, Methodology. **Fen Liu:** Investigation. **Yan Wang:** Investigation. **Tong Dai:** Data curation. **Xia Yang:** Investigation. **Qinyu Yang:** Data curation. **Pengcheng Huang:** Funding acquisition, Supervision, Writing – review & editing. **Chunhua Tu:** Funding acquisition, Project administration, Supervision, Writing – review & editing.

## Declaration of competing interest

The authors declare that they have no known competing financial interests or personal relationships that could have appeared to influence the work reported in this paper.

## Data Availability

Data will be made available on request.
