# Supplementary material for: Mapping of uterine-related neurons in central nervous system of mice by trans-synaptic tracing with pseudorabies virus
Source: Biochem Biophys Rep. 2026 May 17;46:102630. doi: 10.1016/j.bbrep.2026.102630 (PMC13197711; doi:10.1016/j.bbrep.2026.102630)
Supplement: Multimedia component 1 [file mmc1.docx]

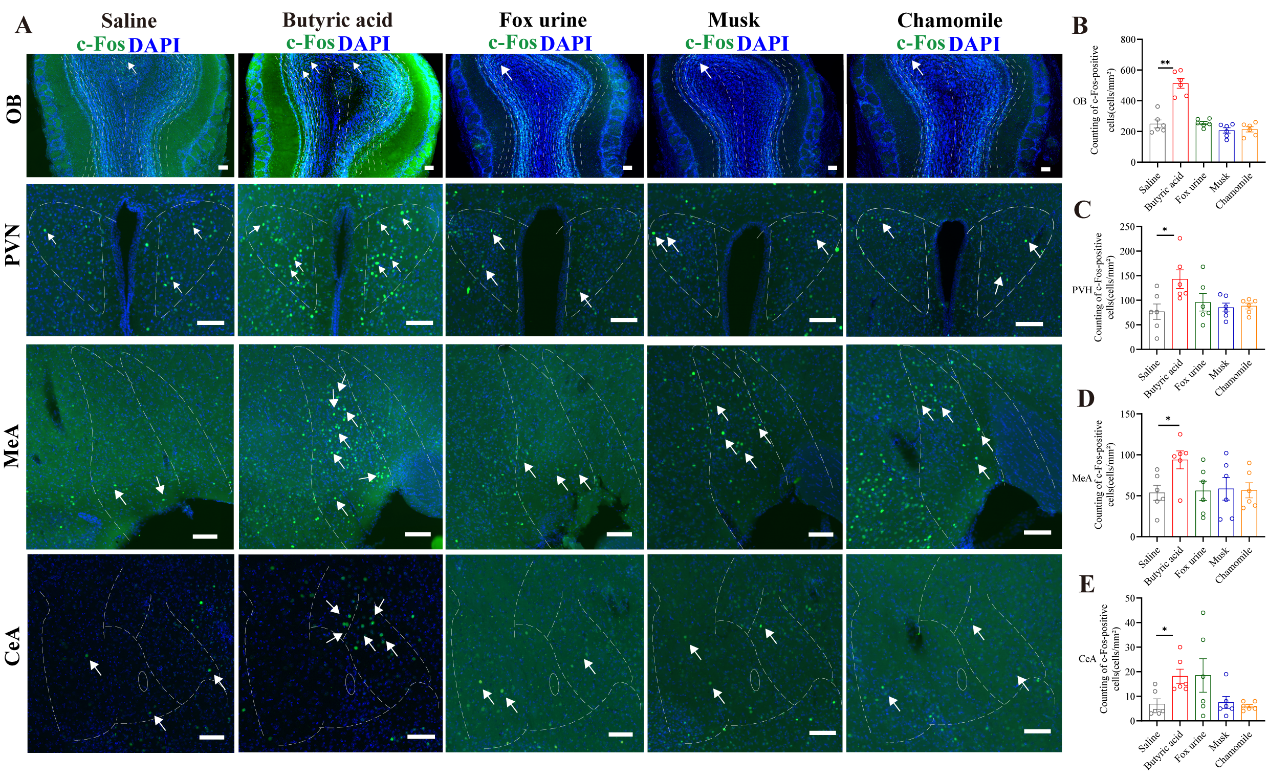


**Supplementary Figure 1. Only BA exposure significantly activates neurons in the OB, PVN, CeA, and MeA brain regions.**

(A) Representative immunofluorescence images of c‑Fos staining in the OB, PVN, MeA, and CeA of mice following exposure to saline, BA, fox urine, muscone, or chamomile, scale bar 100 μm.

(B–E) Histograms of c-Fos expression in OB, PVN, MeA, and CeA (n = 6), **p* < 0.05, ***p* < 0.01, two-way ANOVA followed by Tukey's post hoc test.
