# Supplementary material for: Mapping of uterine-related neurons in central nervous system of mice by trans-synaptic tracing with pseudorabies virus
Source: Biochem Biophys Rep. 2026 May 17;46:102630. doi: 10.1016/j.bbrep.2026.102630 (PMC13197711; doi:10.1016/j.bbrep.2026.102630)
Supplement: Multimedia component 2 [file mmc2.docx]

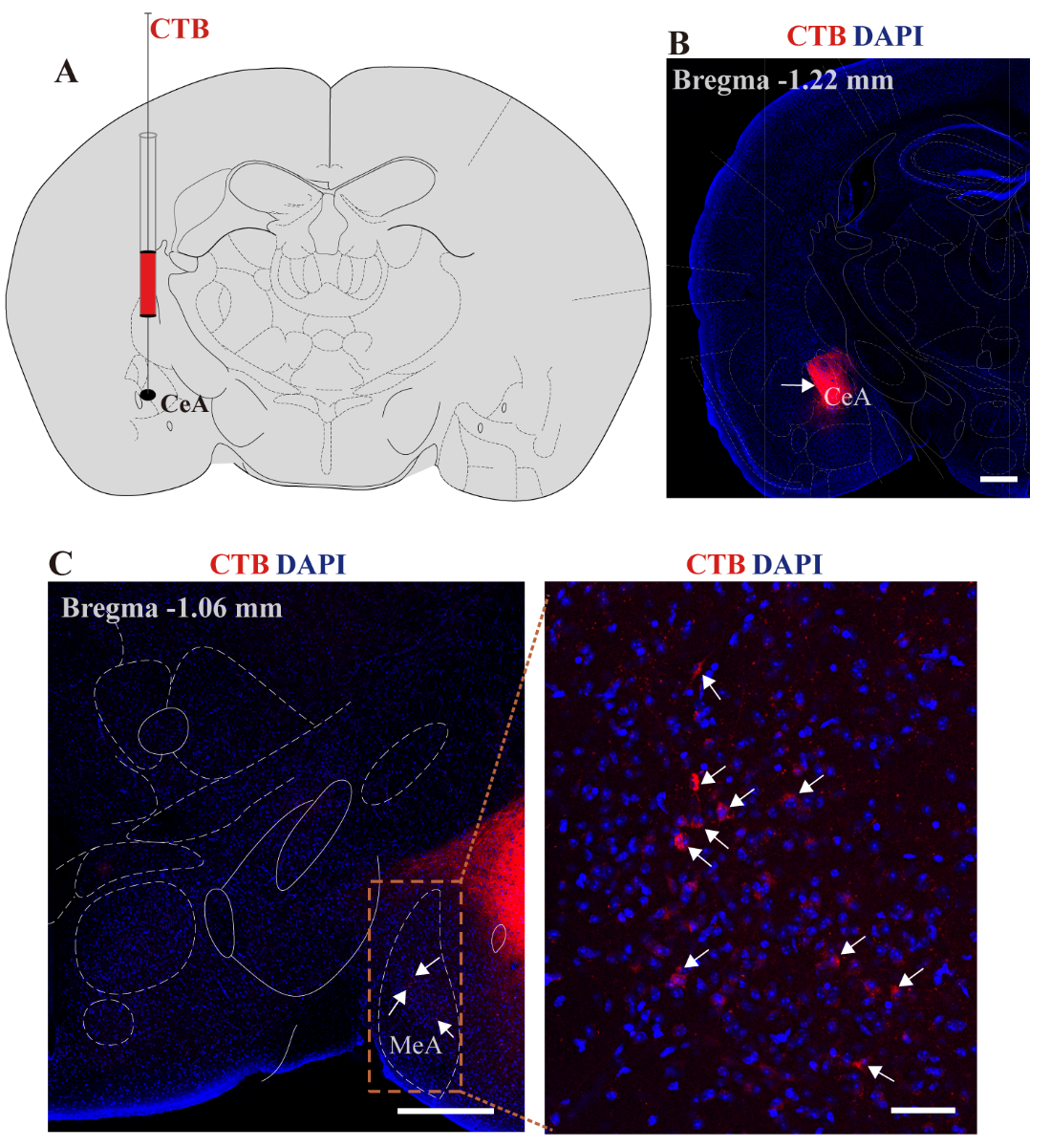


**Supplementary Figure 2. Projective neurons were observed in MeA following the CTB injected into the CeA.**

(A) Schematic diagram of CTB injection into the CeA.

(B) Representative image of CTB555 injection site in the CeA, scale bar, 500 μm.

(C) Representative image shows no CTB555-labeled neurons in the MeA, scale bar, 500 μm. The right panel shows a magnified view. Scale bar, 50 μm.
