# Supplementary material for: Mapping of uterine-related neurons in central nervous system of mice by trans-synaptic tracing with pseudorabies virus
Source: Biochem Biophys Rep. 2026 May 17;46:102630. doi: 10.1016/j.bbrep.2026.102630 (PMC13197711; doi:10.1016/j.bbrep.2026.102630)
Supplement: Multimedia component 3 [file mmc3.docx]

| **Supplementary Table 1. Comparison of neuroanatomical results from PRV retrograde tracing between mice and rats (uterus-to-brain pathway)** | |
| --- | --- |
| Mouse study (this work) | Rat studies (Papka et al., 1998; Lee and Erskine, 2000) |
| Injection site: myometrium of the uterine horn (unilateral). | Injection site: myometrium of uterine cervix/body. |
| Viral tool: PRV-CAG-EGFP, direct fluorescent protein reporter. | Viral tool: PRV Bartha strain, detected by immunohistochemistry. |
| Time-dependent: spinal cord to medulla to hypothalamus. | Time-dependent: spinal cord to medulla to hypothalamus. |
| Key relay nuclei of the spinal cord (IML), brainstem (NTS, RVL, PAG, MPB, LC), and hypothalamus (PVN). | Key relay nuclei of the spinal cord (IML), brainstem (NTS, RVL, PAG, MPB, LC), and hypothalamus (PVN). |
| Barrington's nucleus: clearly labeled, but not to the degree described as "extremely dense". | Barrington's nucleus: described as "extremely densely". |
| SCH: no significant labeling observed. | SCH: reported. |
| Amygdala and related cortices: dense, ipsilaterally predominant labeling observed in APir, LEnt, and IPAC at late infection times. | Amygdala and related cortices: mentioned but not emphasized; No reports of dense labeling in APir, LEnt, or IPAC. |
| Clear ipsilateral (injection-side) predominance of labeling was observed in regions like APir, LEnt, and IPAC. | Ipsilateral predominance driven by the injection side was not specifically analyzed or reported. |
| Table note: IML, intermediolateral nucleus; NTS, nucleus of the solitary tract; RVL, rostral ventrolateral medulla; PAG, periaqueductal gray; MPB, medial parabrachial nucleus; LC, locus coeruleus; PVN, paraventricular nucleus; APir, amygdalopiriform area; LEnt, lateral entorhinal cortex; IPAC, interstitial nucleus of the posterior limb of the anterior commissure; SCH, suprachiasmatic nucleus; PRV, pseudorabies virus; PRV-CAG-EGFP, pseudorabies virus with CAG promoter driving EGFP expression. | |
